# Consumption of Health-Related Videos and Human Papillomavirus Awareness: Cross-Sectional Analyses of a US National Survey and YouTube From the Urban-Rural Context

**DOI:** 10.2196/49749

**Published:** 2024-01-15

**Authors:** Ashvita Garg, Alan G Nyitray, James R Roberts, Nicholas Shungu, Kenneth J Ruggiero, Jessica Chandler, Haluk Damgacioglu, Yenan Zhu, Naomi C Brownstein, Katherine R Sterba, Ashish A Deshmukh, Kalyani Sonawane

**Affiliations:** 1 Medical University of South Carolina Charleston, SC United States; 2 Hollings Cancer Center Charleston, SC United States; 3 Medical College of Wisconsin Milwaukee, IL United States; 4 Medical College of Wisconsin Cancer Center Milwaukee, IL United States; 5 Applications Center for Healthful Lifestyles Charleston, SC United States

**Keywords:** awareness, health awareness, health information, health videos, HINTS, HPV vaccine, HPV, information behavior, information behaviors, information seeking, online information, reproductive health, rural, sexual health, sexually transmitted, social media, STD, STI, urban, video, videos, YouTube

## Abstract

**Background:**

Nearly 70% of Americans use the internet as their first source of information for health-related questions. Contemporary data on the consumption of web-based videos containing health information among American adults by urbanity or rurality is currently unavailable, and its link with health topic awareness, particularly for human papillomavirus (HPV), is not known.

**Objective:**

We aim to describe trends and patterns in the consumption of health-related videos on social media from an urban-rural context, examine the association between exposure to health-related videos on social media and awareness of health topics (ie, HPV and HPV vaccine), and understand public interest in HPV-related video content through search terms and engagement analytics.

**Methods:**

We conducted a cross-sectional analysis of the US Health Information National Trends Survey 6, a nationally representative survey that collects data from civilian, noninstitutionalized adults aged 18 years or older residing in the United States. Bivariable analyses were used to estimate the prevalence of consumption of health-related videos on social media among US adults overall and by urbanity or rurality. Multivariable logistic regression models were used to examine the association between the consumption of health-related videos and HPV awareness among urban and rural adults. To provide additional context on the public’s interest in HPV-specific video content, we examined search volumes (quantitative) and related query searches (qualitative) for the terms “HPV” and “HPV vaccine” on YouTube.

**Results:**

In 2022, 59.6% of US adults (152.3 million) consumed health-related videos on social media, an increase of nearly 100% from 2017 to 2022. Prevalence increased among adults living in both urban (from 31.4% in 2017 to 59.8% in 2022; *P*<.001) and rural (from 22.4% in 2017 to 58% in 2022; *P*<.001) regions. Within the urban and rural groups, consumption of health-related videos on social media was most prevalent among adults aged between 18 and 40 years and college graduates or higher-educated adults. Among both urban and rural groups, adults who consumed health-related videos had a significantly higher probability of being aware of HPV and the HPV vaccine compared with those who did not watch health videos on the internet. The term “HPV” was more frequently searched on YouTube compared with “HPV vaccine.” Individuals were most commonly searching for videos that covered content about the HPV vaccine, HPV in males, and side effects of the HPV vaccine.

**Conclusions:**

The consumption of health-related videos on social media in the United States increased dramatically between 2017 and 2022. The rise was prominent among both urban and rural adults. Watching a health-related video on social media was associated with a greater probability of being aware of HPV and the HPV vaccine. Additional research on designing and developing social media strategies is needed to increase public awareness of health topics.

## Introduction

Access to the internet is deemed to be an important social determinant of health [1,2]. Nearly 80% of Americans seek health information on the web, and almost 70% are reportedly using the internet as their first source of information for health-related questions [3-5]. Although data suggest that urban-rural disparities in access to the internet continue to persist, with the advent and wide availability of broadband cellular network technology, the “digital divide” is likely to narrow in the future, making web-based health content more accessible to rural and underserved communities [6,7]. There are a plethora of avenues (blogs, informational webpages, social media, and health support groups) and various formats (links, status updates, polls, pictures, and videos) for sharing health information. Literature suggests that user engagement with web-based health content is generally greater if it includes a visual component such as a photo or video [8-11]. Trials evaluating digital interventions that included educational or health-promotional videos have reported positive outcomes (increased knowledge, intentions, and actions in individuals) for disease prevention (human papillomavirus [HPV] vaccination, and cancer, HIV, and fall prevention) [12-15].

The popularity of web-based video-sharing platforms, such as YouTube, has grown in recent years. While internet accessibility has been extensively compared between urban and rural geographies, contemporary US data quantifying geographical differences in exposure to health-related videos on social media are not available. It is important to quantify these data in the context of “place,” as the expansion of the cellular network has improved accessibility to health information available on the web in urban as well as rural areas. In this study, we estimate the prevalence of watching health-related videos among rural and urban American adults using the nationally representative Health Information National Trends Survey (HINTS). Furthermore, according to the structural model of health communication, urbanity or rurality is a key social determinant that impacts health outcomes (ie, knowledge, comprehension, health beliefs, health behaviors, and prevention), and its effect is moderated by health media exposure [16,17]. Although empirical research has established that individuals from rural communities have poor health awareness and experience worse disease outcomes [18-21], the intersection of urbanity or rurality with health media consumption and its association with health awareness has not been established using a representative sample of urban and rural adults. We leveraged the HPV (a very common infection that can cause genital warts and cancers) awareness questionnaire from the survey to examine whether watching health-related videos decreases (or increases) the probability of HPV awareness among urban- and rural-dwelling adults. In addition, we evaluated YouTube (the largest video-sharing website) data alongside the survey data to provide context on the public’s interest in HPV-related videos.

## Methods

### Data Sources

This study used the 2022 HINTS 6 data (administered between March and November 2022) to derive the latest and most up-to-date estimates [22]. The 2017 HINTS 5 was used to compute the change in prevalence only. The survey represents a nationally representative sample, and it is conducted by the US National Cancer Institutes (NCI) to monitor changes in the rapidly evolving fields of health communication and health information technology. Data are collected from civilian, noninstitutionalized adults aged 18 years or older residing in the united States. Details regarding the survey design and methodology are available elsewhere [22]. Data on the popularity and viewership of web-based videos were extracted from 2 publicly available sources: the Google Trends and the YouTube Data Application Programming Interface (API; version 3), respectively [23,24]. Google Trends provides access to a largely unfiltered sample of search requests made to the Google search engine. Data are anonymized, categorized by topic for a search query, and can be restricted to provide searches specific to social media platforms. Public interest in a particular topic from around the globe and from more granular areas (country, state, and metropolitan urban areas) is available. Google Trends provides volume-standardized scores of keyword searches; that is, the highest volume of searches originating for a given spatial unit and time is assigned a value of 100, and all subsequent volumes are assigned relative scores. For instance, a score of 50 means that the term was half as popular, and a score of 0 means there was not enough data. The YouTube Data API is the official public-facing API of the web-based video-sharing and social media platform YouTube. The API is used in this study to capture the viewership and engagement of the public with web-based video content on HPV topics. The API provides researchers or users access to the YouTube data server [24]. Researchers can submit queries for topics of interest through the interface using function calls to access information (views, likes, comments, captions, subscribers, and sponsors) for YouTube videos, channels, or playlists.

### Variables of Interest

Outcomes of interest were watching a health-related video on social media (primary outcome) and HPV topic awareness (secondary outcome). The primary outcome was a self-reported indicator of watching health-related web-based videos (“yes” or “no”). Participants were asked, “In the last 12 months, how often did you watch a health-related video on a social media site (for example, YouTube)?” Participants who watched health-related videos on social media in the past year were coded as “yes,” and those who reported never watching health videos were coded as “no.” Awareness of HPV as a health topic was captured through HPV awareness (Have you ever heard of HPV?) and HPV vaccine awareness (Before today, have you ever heard of the cervical cancer vaccine or HPV shot?) questions with dichotomous response options (“yes” or “no”). The rurality variable was based on the Rural-Urban Commuting Area (RUCA) codes; areas with RUCA codes ≥4 were identified as rural.

We identified the following demographic variables: age (18-40 years, 41-60 years, 61-70 years, and ≥71 years), sex assigned at birth (female or male), and race or ethnicity (non-Hispanic White, non-Hispanic Black, Hispanic, and others). We also identified other sociodemographic variables: education (less than high school, high school graduate, some college, and college graduate or higher), annual household income (less than US $50,000, US $50,000-US $74,999, US $75,000-US $99,999, US $100,000-US $199,999, and ≥US $200,000), and cancer history.

To capture the public’s interest in HPV-related content, we identified patterns in the search for videos containing HPV information shared on the YouTube platform in the 3 years (2019-2021) preceding the HINTS 6. The standardized search volume overall, by each month, and by metropolitan statistical area were identified for the term “HPV.” To help put the search volume about the HPV topic into perspective and get a sense of relative size, we reported data on the HPV vaccine as a comparison term. We restricted the data originating from the United States to searches that were determined to be queried in the context of health. The top related queries, that is, terms that users typed when searching for HPV or HPV vaccine–related videos on YouTube, were identified. The YouTube data API was searched for the keyword terms “HPV” and “HPV vaccine,” restricted to videos published before December 2022. The API identified the top 500 videos tagged “HPV” or “HPV vaccine” and provided metrics on views (ie, the total number of views) and engagement (ie, the total number of likes and comments) on those videos.

### Statistical Analyses

Descriptive statistics were used to present the sociodemographic characteristics of the final sample by urbanity or rurality and by web-based health video consumption. Bivariable analyses were performed to estimate and compare weighted proportions for HPV and HPV vaccine awareness by consumption of health-related videos on social media; differences between groups (urban vs rural) were tested using the Wald *F* statistic, accounting for multiple comparisons. Similarly, we compared HPV and HPV vaccine awareness between those who watched versus those who did not watch health-related videos among the urban and rural groups using the Wald *F* statistic. Multivariable logistic regression models were constructed to estimate the predicted probability (PP) and difference in predicted probability (dPP) for HPV and HPV vaccine awareness among those who watched health-related videos, compared with those who did not watch health-related videos as the reference group. Standardized search volumes and frequency of engagement (views, likes, and comments) were used to describe patterns in query search and audience engagement with HPV- and HPV vaccine–related videos on YouTube. We used PROC SURVEY procedures to adjust for the complex survey design and weights. All HINTS analyses were conducted using SAS (version 9.4; SAS Institute) statistical software. We used the R 4.2.1 (R Project for Statistical Computing) programming language to derive estimates from the public YouTube data API.

### Ethical Considerations

This study was deemed exempt from review by the institutional review board at the Medical University of South Carolina (Pro00128988). All respondents provided electronic consent and received a US $10 Amazon e-gift card from HINTS after completing the survey.

## Results

A total of 6252 participants (5442 urban and 811 rural) were identified from 2022 HINTS 6 (Figure S1 and Table S1 in Multimedia Appendix 1). Information on watching web-related videos was available for 6158 participants (5358 urban and 800 rural). In 2022, 59.6% (3473/6158) of the participants, representative of an estimated 152.3 million Americans, watched health-related videos on social media (Multimedia Appendix 2).

The prevalence of watching health-related videos increased by nearly 100% (from 30.1% to 59.6%, *P*<.001) between 2017 and 2022. The increase in prevalence in both urban (from 31.4% in 2017 to 59.8% in 2022; *P*<.001) and rural (from 22.4% in 2017 to 58.0% in 2022; *P*<.001) US adults was statistically significant. The 9%-point difference between urban versus rural adults who watched videos observed during 2017 (*P*=.02) reduced to 1.5%-point in 2022 (*P*=.52).

Table 1 describes the prevalence of watching health-related social media videos among urban and rural adults across sociodemographic strata**.** In the urban group, watching health-related videos on social media was most prevalent among participants in the 18-40 years (1018/1329, 72.5%) age group; for “others,” it was 74.9% (303/427); for college graduates or higher-educated adults, it was 69.3% (1563/2427); and for individuals living in households with annual income ≥US $200,000, it was 65.8% (294/453). The prevalence in the rural group was higher among individuals in the 18-40 years age group (100/129, 76.7%) and was highest for college graduates or higher-educated adults (171/282, 68.3%). A table describing the characteristics of participants who watched versus those who did not watch health-related videos on the web is presented in Table S2 in Multimedia Appendix 1.

**Table 1:** Prevalence of watching health-related videos on social media among urban and rural adults by sociodemographic strata, 2022 Health Information National Trends Survey (HINTS) 6.

Characteristics^a^		Urban^b^ (n=5,358)					*P* value			Rural^b^ (n=800)						*P* value	
		Watched (n=3069)		Did not watch (n=2289)					Watched (n=404)				Did not watch (n=396)				
		Unweighted, n	Weighted, N (%)	Unweighted, n	Weighted, N (%)				Unweighted, n			Weighted, N (%)	Unweighted, n	Weighted, N (%)			
**Age (years)**								<.001									<.001
	18-40	1018	59,860,846 (72.5)	311	22,650,452 (27.5)				100			6,214,335 (76.7)	29	1,884,450 (23.3)			
	41-60	1064	48,666,504 (61.7)	606	30,158,575 (38.3)				152			7,980,602 (66.4)	96	4,039,696 (33.6)			
	61-70	589	16,077,457 (48.7)	596	16,949,155 (51.3)				88			2,543,072 (42)	121	3,510,612 (58)			
	≥71	376	8,666,125 (31.5)	731	18,823,670 (68.5)				59			1,417,340 (28.6)	138	3,536,450 (71.4)			
**Sex**								.24									.62
	Female	1723	64,714,544 (61.1)	1296	41,153,419 (38.9)				254			8,987,216 (58.4)	220	6,413,763 (41.6)			
	Male	1124	60,378,555 (58.2)	876	43,323,733 (41.8)				129			8,193,155 (56.1)	162	6,415,736 (43.9)			
**Race or ethnicity**								<.001									.07
	Non-Hispanic White	1407	66,027,091 (54.7)	1232	54,622,063 (45.3)				279			12,302,142 (56.4)	269	9,527,994 (43.6)			
	Non-Hispanic Black	487	16,301,275 (68.3)	312	7,572,056 (31.7)				36			672,640 (42.6)	39	906,835 (57.4)			
	Hispanic	589	24,522,106 (65.4)	353	12,987,442 (34.6)				24			1,346,027 (68.5)	20	618,243 (31.5)			
	Others	303	16,539,325 (74.9)	124	5,539,062 (25.1)				25			2,227,711 (75.2)	16	733,214 (24.8)			
**Education**								<.001									.008
	Less than high school	121	5,430,530 (40)	197	8,150,012 (60)				15			1,098,344 (43.1)	40	1,449,996 (56.9)			
	High school graduate	369	20,727,370 (49.2)	475	21,365,065 (50.8)				75			4,550,256 (49.4)	129	4,668,751 (50.6)			
	Some college	798	48,663,876 (59.6)	634	33,008,983 (40.4)				122			6,536,964 (59.1)	103	4,517,706 (40.9)			
	College graduate or higher	1563	49,263,948 (69.3)	864	21,842,753 (30.7)				171			4,791,613 (68.3)	111	2,228,229 (31.7)			
**Annual household income (US $)**								.002									.40
	0-49,999	1192	45,185,976 (53.8)	1149	38,727,795 (46.2)				200			8,668,914 (53.5)	248	7,549,338 (46.5)			
	50,000-74,999	524	23,538,835 (60.3)	347	15,529,334 (39.7)				62			2,557,549 (54.1)	61	2,167,305 (45.9)			
	75,000-99,999	409	17,362,615 (60.4)	272	11,371,208 (39.6)				54			3,144,168 (68.8)	35	1,426,496 (31.2)			
	100,000-199,999	646	31,712,428 (65.7)	357	16,548,180 (34.3)				67			2,869,756 (65.2)	39	1,528,962 (34.8)			
	≥200,000	294	14,974,547 (65.8)	159	7,775,563 (34.2)				19			754,263 (64.5)	11	414,392 (35.5)			
Cancer history (yes)		329	9,802,776 (48.8)	411	10,298,077 (51.2)	<.001			65			1,989,773 (49.6)	75	2,020,963 (50.4)	.21		

^a^Sociodemographic characteristics were self-reported by participants. Information on age (n=98), sex (n=410), race or ethnicity (n=687), education (n=404), income (n=17), and cancer history (n=370) were missing for some participants.

^b^Urban-rural designations were identified from Rural-Urban Commuting Area Code (RUCA) codes. RUCA ≥4 were classified as rural based on the US Census Bureau and the Rural Health Information Hub.

Awareness of HPV and the HPV vaccine was higher in those who watched versus those who did not watch health-related YouTube videos among both urban and rural groups (Multimedia Appendix 3). Within the urban group, awareness of HPV was 26.1% higher (*P*<.001) and that of the HPV vaccine was 15.2% (*P*<.001) higher in those who reportedly watched health-related videos. Similarly, in the rural group, awareness of HPV (33.5% higher; *P*<.001) and HPV vaccine (23.7% higher; *P*<.001) was greater in those who watched a health-related video in the past 12 months than in those who did not watch videos. The findings were consistent with adjusted estimates from multivariable models (Table S3 in Multimedia Appendix 1). In the rural group, probability of being aware of the HPV (difference in predicted probability [dPP] 21.2%, 95% CI 18.5%-24.0%) and the HPV vaccine (dPP 14.6%, 95% CI 11.7%-17.6%) was greater for individuals who watched a health-related video compared with those who do not watch health videos on the internet. Similarly, the probability of being aware of the HPV (dPP 18.3%, 95% CI 17.1%-19.5%) and the HPV vaccine (dPP 11.8%, 95% CI 10.4%-13.2%) was greater among urban adults who watched versus those who did not watch health-related videos on social media.

**Figure 1:**
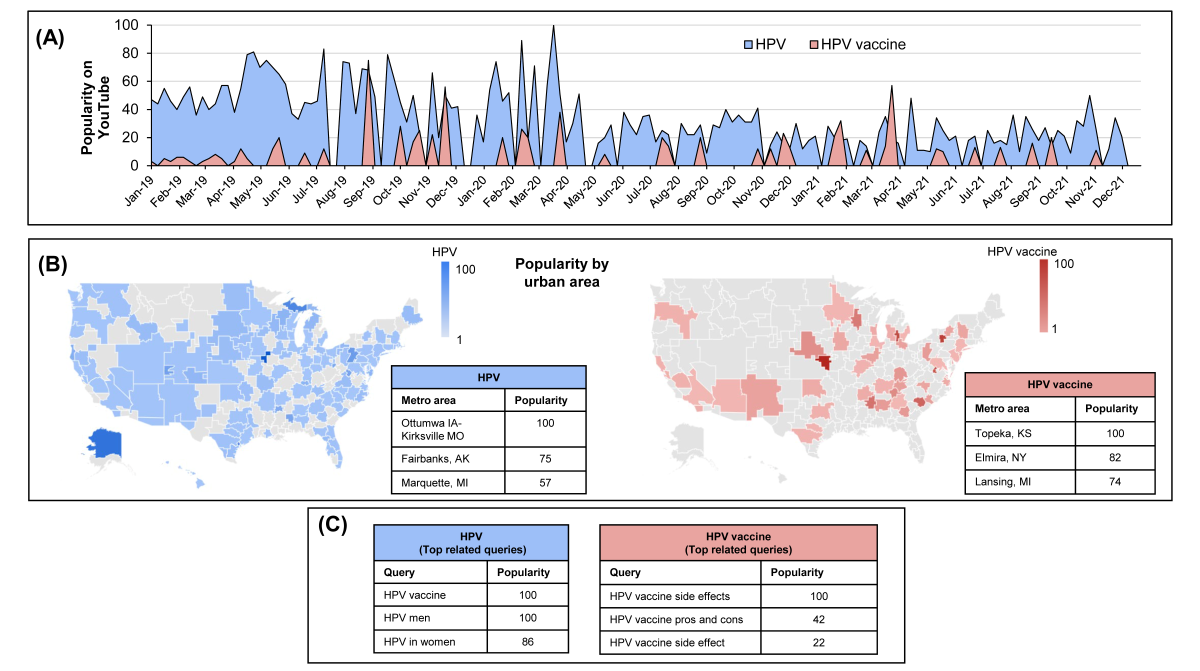
Patterns in seeking human papillomavirus (HPV) and HPV vaccine content on YouTube. Panels (A-C) illustrate the patterns in HPV (blue) and HPV vaccine (red) query searches on YouTube in the years 2019-2021 preceding the 2022 Health Information National Trends Survey (HINTS) 6. (A) This illustrates time trends in query search standardized relative to peak popularity during 2019-2021. (B) This shows popularity across large urban metropolitan areas in the United States. (C) This lists the top 3 most popular terms users searched with “HPV” and “HPV vaccine.” Trends in query search on YouTube were examined using Google Trends restricted to 3 years before the 2022 HINTS 6 (ie, 2019-2021).

Figure 1 illustrates space-time patterns in web-based searches for HPV and HPV vaccine–related videos on YouTube preceding the 2022 HINTS 6. Users predominantly searched for HPV content using the keyword “HPV” and less frequently using the term “HPV vaccine,” as suggested by the average standardized search score of 31 and 5, respectively, over the 3 years. Across all US urban areas, the highest relative volume of searches for HPV and HPV vaccine content on YouTube originated from Ottumwa, IA; Kirksville, MO; and Topeka, KS, respectively. The most popular queries users searched in combination with “HPV” were “HPV vaccine” and “HPV men,” and for “HPV vaccine,” the related query “HPV vaccine side effects” was most popular. The top 500 videos related to HPV garnered a total of 54.8 million views, 769,276 likes, and 68,933 comments since publication, and the top 500 HPV vaccine videos garnered a total of 13.6 million views, 77,813 likes, and 25,277 comments (Figure S2 in Multimedia Appendix 1).

## Discussion

This is the first study, to our knowledge, that estimated the prevalence of watching health videos on social media among urban and rural US adults. Overall, more than one-half of American adults in 2022 reported that they watched health-related videos. The prevalence of watching videos was not statistically significantly different between urban (3069/5358, 59.8%) and rural (404/800, 58.0%) residents in the United States. Awareness of HPV topics, including awareness of both the HPV and the HPV vaccine, was statistically significantly higher (all *P*<.001) among participants who watched health-related videos compared with those who did not watch videos, regardless of urbanity or rurality. Our data shows that the number of US individuals watching web-based videos that contain health information increased dramatically during 2017-2022 (100% increase) in the United States, and HPV awareness was relatively higher in individuals who watched health content on web-based platforms compared with individuals who did not watch web-based health-related videos.

Social media platforms have become a prominent source of exposure to health content created by medical experts or organizations as well as nonprofessionals. The number of social media platforms and their users has grown exponentially. YouTube alone is reportedly used by over 84% of urban and 74% of rural US adults [25]. We found that searches for videos pertaining to “HPV men,” “HPV women,” “HPV vaccine,” “HPV vaccine side effects,” and “HPV pros and cons” were most commonly on YouTube. These patterns in searches suggest that individuals might be seeking videos to gain an understanding of HPV infection in men (men have relatively lower knowledge about HPV than women) [26] as well as understand the benefits versus harms of getting the HPV vaccine (recent studies report safety concerns are increasingly contributing towards HPV vaccine hesitancy) [27,28]. The millions of views and positive engagement metrics on YouTube illustrate that sharing and seeking information on social media websites have become deeply rooted, regardless of place. The readily available, easily accessible web-based health information ecosystem has altered the ways in which individuals, communities, and societies are exposed to health information on a day-to-day basis.

At an individual level, the nature of information (favorable or unfavorable) can alter perceptions and actions either positively or negatively. For example, exposure to positive information regarding the HPV vaccine on social media increased the likelihood of having favorable views regarding the HPV vaccine in one study [29]. In contrast, encounters with antivaccine content were linked with a decrease in HPV vaccine uptake, mediated through increased vaccine hesitancy [30]. Previous studies report that the majority of HPV vaccination videos on social media tend to be neutral or negative in tone [31,32]. Similar to evidence from individual-level studies, HPV vaccine information exposure at the community or societal level is reportedly positively associated with vaccination rates. In Australia, regional HPV vaccine coverage in females (*R*=.75, 0.49-0.88) and males (*R*=.76, 0.51-0.89) was found to be strongly associated with area-level exposure to HPV vaccine tweets [33]. Findings were similar in a US-based study; HPV vaccination information exposure on Twitter (subsequently rebranded X) across the 50 states contributed to 68% of the variance in HPV vaccine coverage among female individuals and 63% among male individuals [34]. These ecological studies indicate that beyond individual-level interventions, social media can also be exploited for social marketing to increase public awareness of health topics.

Community and societal exemplars of HPV vaccination are also worth mentioning. A few years ago, the country of Denmark saw a sharp decline (reduced to 49.6%) in HPV vaccine coverage due to widespread vaccine misinformation. In response, the Danish Health Authority, the Danish Cancer Society, and the Danish Medical Association launched a strategic national campaign “Stop HPV–stop cervical cancer” (Stop HPV–stop livmoderhalskræft) to disseminate informational videos through social media [35,36]. The campaign was highly successful in combating HPV vaccine hesitancy. Engagement and the number of positive comments regarding the HPV vaccine reached nearly 75% after the campaign launch [35]. The campaign eventually led to the full recovery (100%) of HPV vaccination rates [36]. Ireland faced similar challenges (coverage dropped from 89.7% to 50%) due to widespread misinformation on HPV vaccination and was able to implement a national campaign to tackle plummeting HPV vaccination rates. The Irish National Immunization Office launched media campaigns that included personal narrative videos strongly supporting HPV vaccination alongside a comprehensive training program for health professionals [37]. Multiple studies show that user engagement is highest with informational health content that includes a visual component, such as a photo or video [8-11]. These exemplars of bringing HPV vaccination back on track provide support for communicating health information in video format to a broad audience, including those living in rural areas who are otherwise hard to reach. Targeted outreach through paid promotions and investment in video content creation will be key to maximizing the impact of health communication in rural communities [38]. Pairing with additional resources (eg, provider communication, enhanced access, and reminders) will be necessary to overcome barriers to the uptake of medical intervention in communities.

Indeed, trials as well as a social media campaign study have reported positive and significant health outcomes (increased knowledge, intentions, and actions in individuals) with video-based educational interventions for cancer prevention [12,39]. In a large multicenter trial, an informational HPV vaccination video intervention directed toward parents increased the odds of their child receiving an HPV vaccine dose (odds ratio 3.07, CI 1.47-6.42; *P*=.003) [39]. Pooled data estimates from a meta-analysis suggest that educational interventions delivered in audio-visual format are effective (risk ratio 1.27, 95% CI 1.15-1.39; 4 studies; 2542 participants) for increasing HPV vaccination [12]. In a social media campaign study that used educational videos for the dissemination of HPV vaccine information, increases in perceived susceptibility (*P*<.01), perceived severity (*P*<.05), and self-efficacy (*P*<.01) were reported in a postcampaign survey [40]. Dissemination of evidence-based and scientifically accurate information through videos will be specifically important for improving HPV topic awareness (overall HPV awareness among urban and rural US adults is reportedly low at 67.2% and 55.8%, respectively) [41] in both urban and rural individuals and might help them make informed decisions regarding HPV vaccination and cancer screenings. This is particularly important in the context of the rapidly rising incidence and burden of cancers caused by HPV in the United States [42-48]. Collectively, in the light of recent evidence highlighting knowledge gaps in the United States regarding susceptibility to HPV and incorrect perceptions regarding the safety and effectiveness of the HPV vaccine [27,28,41,49], this population-based study and previous research provide important evidence in support of using informational videos for increasing health topic awareness in the public.

The limitations of this study should be carefully considered when interpreting the findings. Exposure to health-related videos and HPV awareness were evaluated in general terms (“yes” or “no”), but the nature of the video content (quality, depth, message framing, and tone) and the level of awareness were not available in the survey. Additionally, the awareness questionnaire in the HINTS 6 was limited to HPV; therefore, we could not examine awareness of other health topics in relation to watching health-related videos on social media websites. Nevertheless, HPV awareness was a reasonable outcome because (1) the entire population of US adults (aged 18 years or older and being sexually active) is considered “at risk” of acquiring HPV infection, unlike topics (medication adherence, smoking, pregnancy, etc) that might be relevant to only certain subgroups; and (2) HPV awareness is low in the United States despite being the most common sexually transmitted infection [26]. While the large nationally representative sample allowed us to examine the correlation between consumption of health-related videos on social media and HPV awareness, the cross-sectional nature and residual confounding from variables not captured in the data preclude making causal interpretations. YouTube data illustrating public interest are abstracted from social media analytics platforms and cannot differentiate multiple views from the same users or discern user duplicates. Ancillary data on public interest in health-related videos were from YouTube (the world’s largest platform for watching and sharing videos). Survey participants might have watched videos on other social media platforms. However, previous studies examining motivations for using social media platforms have reported “information seeking” as one of their main motivations for watching YouTube content [50,51]. In comparison, in previous studies, the underlying motives for the use of other social platforms were not associated with information seeking (Instagram for “surveillance or knowledge about others;” Facebook and Twitter for “social interaction” and “social connection;” Pinterest for “fashion;” and Reddit for “socializing and community building”) [52-56]. Finally, the urban versus rural dichotomy in this study presents a simplified assessment of exposure-outcome. This relationship should be further explored over the urban-rural continuum in future studies.

In conclusion, more than one-half of urban and rural US adults in this nationally representative study watched health-related videos on social media websites. Exposure to videos containing health information was associated with greater HPV topic awareness. Health communication through informational videos should be further researched as a strategy for increasing health topic awareness in clinic and community settings.

## Appendix

Multimedia Appendix 1 Consumption of health-related videos and HPV awareness: Cross-sectional Analyses of a US national survey and YouTube from the urban-rural context.

Multimedia Appendix 2 Prevalence of watching health-related videos on social media in HINTS 5 (2017) versus HINTS 6 (2022).* The figure illustrates the sample size (unweighted N), population size ( weighted N), and prevalence for consumption of health-related videos on social media among US adults. Abbreviations: HINTS, Health Information National Trends Survey **P* values are for survey design-adjusted Wald F test.

Multimedia Appendix 3 Awareness of HPV and HPV vaccine among urban and rural adults by online health video consumption, 2022 HINTS 6. * The figure illustrates the awareness of HPV and HPV vaccine among urban and rural US adults by consumption by online health video consumption. Abbreviations: HPV, Human papillomavirus **P* values are for survey design-adjusted Wald F test.

## Abbreviations

API: Application Programming Interface
dPP: difference in predicted probability
HINTS: Health Information National Trends Survey
HPV: human papillomavirus
NCI: National Cancer Institutes
PP: predicted probability
RUCA: Rural-Urban Commuting Area
