# Supplementary material for: Consumption of Health-Related Videos and Human Papillomavirus Awareness: Cross-Sectional Analyses of a US National Survey and YouTube From the Urban-Rural Context
Source: J Med Internet Res. 2024 Jan 15;26:e49749. doi: 10.2196/49749 (PMC10825763; doi:10.2196/49749)
Supplement: Multimedia Appendix 1 [file jmir_v26i1e49749_app1.docx]

**SUPPLEMENT**

**Consumption of health-related videos and HPV awareness: Cross-sectional Analyses of a US national survey and YouTube from the urban-rural context**

**Figure S1.** Sample flow.

**
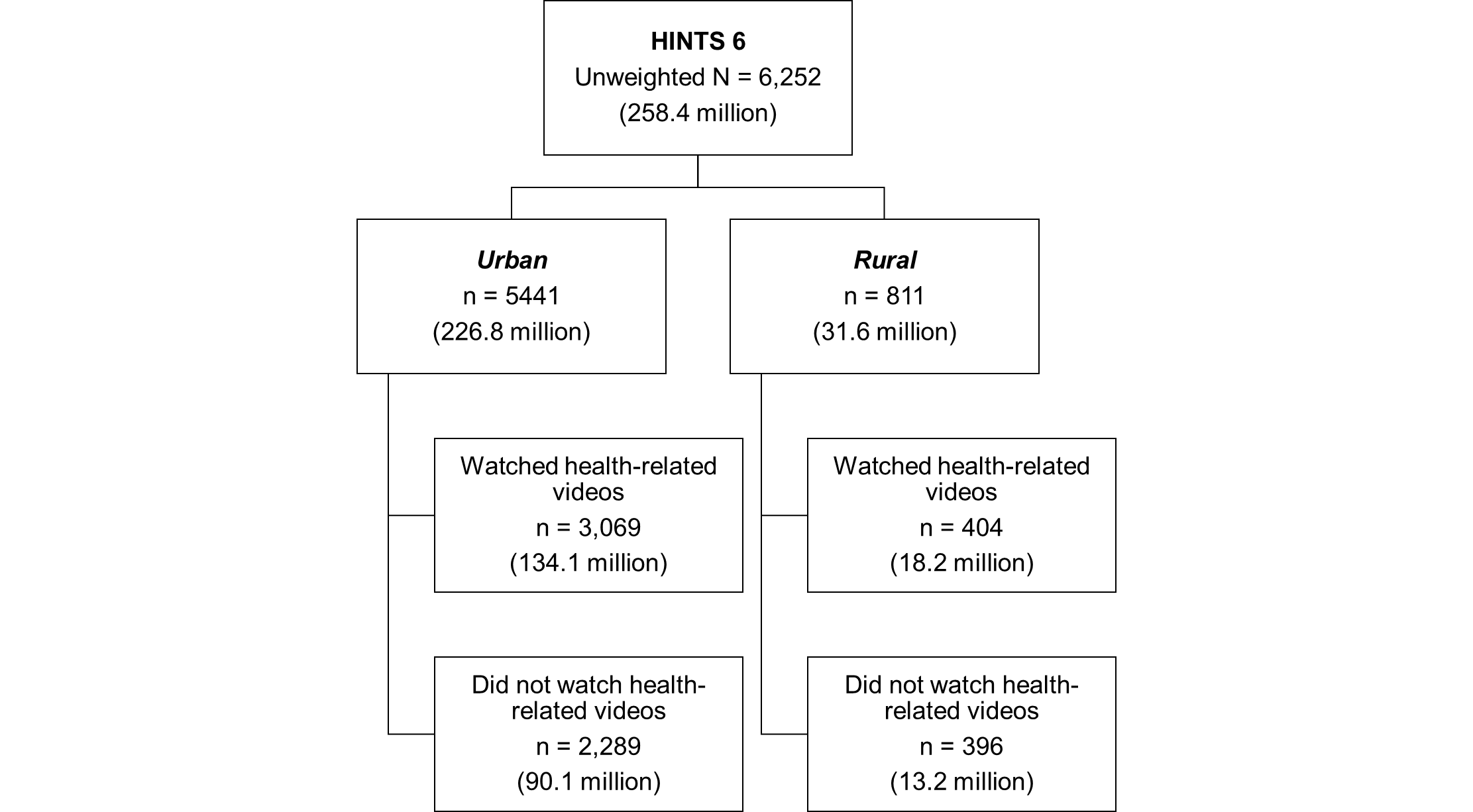
**

**Abbreviations:** HINTS, Health Information National Survey

Notes: Urban-Rural designation was identified from RUCA codes. RUCA 4 and above were classified as rural based on US Census Bureau (<https://www2.census.gov/geo/pdfs/reference/ua/Census_UA_2020FAQs_Feb2023.pdf>) and the rural health information hub (<https://www.ruralhealthinfo.org/topics/what-is-rural>). Information on watching health-related online videos was missing for 83 urban and 11 rural participants.

**Table S1.** Characteristics HINTS 6 participants (overall, urban, and rural).

| Characteristics^*^ | Overall  N=6,252 | Urban  n=5,441 | Rural  n=811 | P  (Urban vs Rural) |
| --- | --- | --- | --- | --- |
|  | *Unweighted n, Weighted n, weighted %* | | |  |
| Age (in years) |  |  |  |  |
| *18-40* | 1,471  91,367,267  35.8 | 1,340  83,208,700  37.1 | 131  8,158,566  26.1 | 0.001 |
| *41-60* | 1,930  91,171,078  35.7 | 1,681  79,101,584  35.3 | 249  12,069,494  38.6 |  |
| *61-70* | 1,414  39,693,218  15.5 | 1,202  33,615,695  15.0 | 212  6,077,524  19.4 |  |
| *71 and older* | 1,339  33,257,678  13.0 | 1,139  28,265,238  12.6 | 200  4,992,440  16.0 |  |
| Sex |  |  |  |  |
| *Female* | 3,535  122,385,407  50.8 | 3,057  106,941,049  50.7 | 478  15,444,359  51.4 | 0.83 |
| *Male* | 2,307  118,697,357  49.2 | 2,015  104,077,961  49.3 | 292  14,619,395  48.6 |  |
| Race/Ethnicity |  |  |  |  |
| *Non-Hispanic White* | 3,203  143,047,138  61.2 | 2,653  121,184,023  59.0 | 550  21,863,115  77.1 | <0.01 |
| *Non-Hispanic Black* | 889  25,682,584  11.0 | 813  24,098,776  11.7 | 76  1,583,808  5.6 |  |
| *Hispanic* | 1,001  39,839,133  17.0 | 957  37,874,863  18.4 | 44  1,964,270  6.9 |  |
| *Other* | 472  25,142,456  10.8 | 431  22,181,530  10.8 | 41  2,960,925  10.4 |  |
| Education |  |  |  |  |
| Less than high school | 387  16,429,329  6.9 | 331  13,869,146  6.6 | 56  2,560,182  8.6 | <0.01 |
| High school graduate | 1,068  51,845,095  21.6 | 863  42,621,359  20.3 | 205  9,223,736  30.9 |  |
| Some college | 1,672  93,286,477  38.9 | 1,447  82,231,807  39.2 | 225  11,054,670  37.0 |  |
| College graduate or higher | 2,721  78,277,597  32.6 | 2,436  71,220,443  33.9 | 285  7,057,154  23.6 |  |
| Annual Household Income ($) | |  |  |  |
| *0-49,999* | 2,856  102,055,956  39.8 | 2,404  85,757,556  38.1 | 452  16,298,399  52.0 | <0.01 |
| *50,000-74,999* | 999  43,853,143  17.1 | 876  39,128,290  17.4 | 123  4,724,853  15.1 |  |
| *75,000-99,999* | 781  33,794,382  13.2 | 688  29,184,902  13.0 | 93  4,609,480  14.7 |  |
| *100,000-199,999* | 1,116  52,939,382  20.6 | 1,007  48,420,991  21.5 | 109  4,518,391  14.4 |  |
| *≥200,000* | 483  23,918,765  9.3 | 453  22,750,110  10.1 | 30  1,168,655  3.7 |  |
| Had cancer |  |  |  |  |
|  | 900  24,527,579  10.1 | 759  20,505,001  9.6 | 141  4,022,577  13.4 | 0.01 |
| Watched a health-related video on social media in the last 12 months | | |  |  |
|  | 3,473  152,327,315  59.6 | 3,069  134,103,437  59.8 | 404  18,223,878  58.0 | 0.53 |

**Abbreviations:** HINTS, Health Information National Survey

*Sociodemographic characteristics were self-reported by participants.

**Urban-Rural designation was identified from Rural-Urban Commuting Area Code (RUCA) codes. RUCA 4 and above were classified as rural based on the US Census Bureau and the rural health information hub.

**Table S2.**  Characteristics of US adults by Urban/Rural status and consumption of health-related videos on social media, HINTS 6.

| Characteristics* | Urban** | | P  (Watched vs Did not watch videos) | | | Rural** | | | P  (Watched vs Did not watch videos) | | P  (Urban vs Rural) |
| --- | --- | --- | --- | --- | --- | --- | --- | --- | --- | --- | --- |
|  | *Watched videos*  *n=3,069* | *Did not watch videos*  *n=2,289* |  |  |  | *Watched videos*  *n=404* | *Did not watch videos*  *n=396* | |  |  |  |
|  | *Unweighted n, Weighted n, weighted %* | | | | | | | | | | |
| Age (in years) |  |  |  | | |  | |  |  | |  |
| 18-40 | 1,018  59,860,846  44.9 | 311  22,650,452  25.6 | <0.001 | | | 100  6,214,335  34.2 | | 29  1,884,450  14.5 | <0.001 | | 0.062 |
| 41-60 | 1,064  48,666,504  36.5 | 606  30,158,575  34.0 |  | | | 152  7,980,602  44.0 | | 96  4,039,696  31.1 |  | |  |
| 61-70 | 589  16,077,457  12.1 | 596  16,949,155  19.1 |  | | | 88  2,543,072  14.0 | | 121  3,510,612  27.1 |  | |  |
| 71 and older | 376  8,666,125  6.5 | 731  18,823,670  21.3 |  | | | 59  1,417,340  7.8 | | 138  3,536,450  27.3 |  | |  |
| Sex |  |  |  | | |  | |  |  | |  |
| *Female* | 1,723  64,714,544  51.7 | 1,296  41,153,419  48.7 | 0.24 | | | 254  8,987,216  52.3 | | 220  6,413,763  50.0 | 0.62 | | 0.90 |
| *Male* | 1,124  60,378,555  48.3 | 876  43,323,733  51.3 |  | | | 129  8,193,155  47.7 | | 162  6,415,736  50.0 |  | |  |
| Race/Ethnicity |  |  |  | | |  | |  |  | |  |
| *Non-Hispanic White* | 1,407  66,027,091  53.5 | 1,232  54,622,063  67.7 | <0.001 | | | 279  12,302,142  74.3 | | 269  9,527,994  80.8 | 0.07 | | <0.001 |
| *Non-Hispanic Black* | 487  16,301,275  13.2 | 312  7,572,056  9.4 |  | | | 36  672,640  4.1 | | 39  906,835  7.7 |  | |  |
| *Hispanic* | 589  24,522,106  19.9 | 353  12,987,442  16.1 |  | | | 24  1,346,027  8.1 | | 20  618,243  5.2 |  | |  |
| *Other* | 303  16,539,325  13.4 | 124  5,539,062  6.9 |  | | | 25  2,227,711  13.5 | | 16  733,214  6.2 |  | |  |
| Education |  |  |  | | |  | |  |  | |  |
| *Less than high school* | 121  5,430,530  4.4 | 197  8,150,012  9.7 | <0.001 | | | 15  1,098,344  6.5 | | 40  1,449,996  11.3 | 0.008 | | 0.013 |
| *High school graduate* | 369  20,727,370  16.7 | 475  21,365,065  25.3 |  | | | 75  4,550,256  26.8 | | 129  4,668,751  36.3 |  | |  |
| *Some college* | 798  48,663,876  39.2 | 634  33,008,983  39.1 |  | | | 122  6,536,964  38.5 | | 103  4,517,706  35.1 |  | |  |
| *College graduate or higher* | 1,563  49,263,948  39.7 | 864  21,842,753  25.9 |  | | | 171  4,791,613  28.2 | | 111  2,228,229  17.3 |  | |  |
| Annual Household Income ($) | | | | | | | | | | | |
| *0-49,999* | 1,192  45,185,976  34.0 | 1,149  38,727,795  43.1 | 0.002 | | | 200  8,668,914  48.2 | | 248  7,549,338  57.7 | 0.399 | | <0.001 |
| *50,000-74,999* | 524  23,538,835  17.7 | 347  15,529,334  17.3 |  | | | 62  2,557,549  14.2 | | 61  2,167,305  16.6 |  | |  |
| *75,000-99,999* | 409  17,362,615  13.1 | 272  11,371,208  12.6 |  | | | 54  3,144,168  17.5 | | 35  1,426,496  10.9 |  | |  |
| *100,000-199,999* | 646  31,712,428  23.9 | 357  16,548,180  18.4 |  | | | 67  2,869,756  15.9 | | 39  1,528,962  11.7 |  | |  |
| *≥200,000* | 294  14,974,547  11.3 | 159  7,775,563  8.6 |  | | | 19  754,263  4.2 | | 11  414,392  3.2 |  | |  |
| Had Cancer |  |  | |  |  | | |  | |  |  |
|  | 329  9,802,776  7.8 | 411  10,298,077  12.1 | | <0.001 | 65  1,989,773  11.7 | | | 75  2,020,963  15.7 | | 0.213 | 0.045 |

**Abbreviations:** HINTS, Health Information National Survey

*Sociodemographic characteristics were self-reported by participants. Information on age(n=98), sex(n=410), race/ethnicity(n=687), education (n=404), income (n=17), and cancer history (n=370) were missing for some participants.

**Urban-Rural designation was identified from Rural-Urban Commuting Area Code (RUCA) codes. RUCA 4 and above were classified as rural based on the US Census Bureau and the rural health information hub.

**Table S3.** Multivariate regression for HPV and HPV vaccine awareness.^*^

| **Group** | **Aware of HPV** | | **Aware of HPV vaccine** | |
| --- | --- | --- | --- | --- |
|  | *Predicted Probability* | *Difference in Predicted Probability* | *Predicted Probability* | Difference in Predicted *Probability* |
|  | *%, (95% CI)* | | | |
| **Overall** |  |  |  |  |
| *Watched videos* | 77.2 (76.6 to 77.7) | 18.1 (16.9 to 19.3) | 69.3 (68.6 to 70.1) | 11.8 (10.4 to 13.2) |
| *Did not watch videos* | 59.0 (58.0 to 60.0) | Ref | 57.6 (56.4 to 58.7) | Ref |
| **Urban** |  |  |  |  |
| *Watched videos* | 78.8 (78.3 to 79.4) | 18.3 (17.1 to 19.5) | 71.2 (70.4 to 71.9) | 11.8 (10.4 to 13.2) |
| *Did not watch videos* | 60.6 (59.5 to 61.6) | Ref | 59.4 (58.2 to 60.5) | Ref |
| **Rural** |  |  |  |  |
| *Watched videos* | 78.3 (77.0 to 79.6) | 21.2 (18.5 to 24.0) | 71.6 (70.0 to 73.3) | 14.6 (11.7 to 17.6) |
| *Did not watch videos* | 57.1 (54.8 to 59.4) | Ref | 57.0 (54.7 to 59.3) | Ref |

**Abbreviations:** HPV, Human papillomavirus

*Logistic regression model adjusted for statistically significantly different characteristics (age, race/ethnicity, education, income, and cancer history)

**Figure S2.** Public interest in HPV- and HPV vaccine-related videos on YouTube^*^.

**
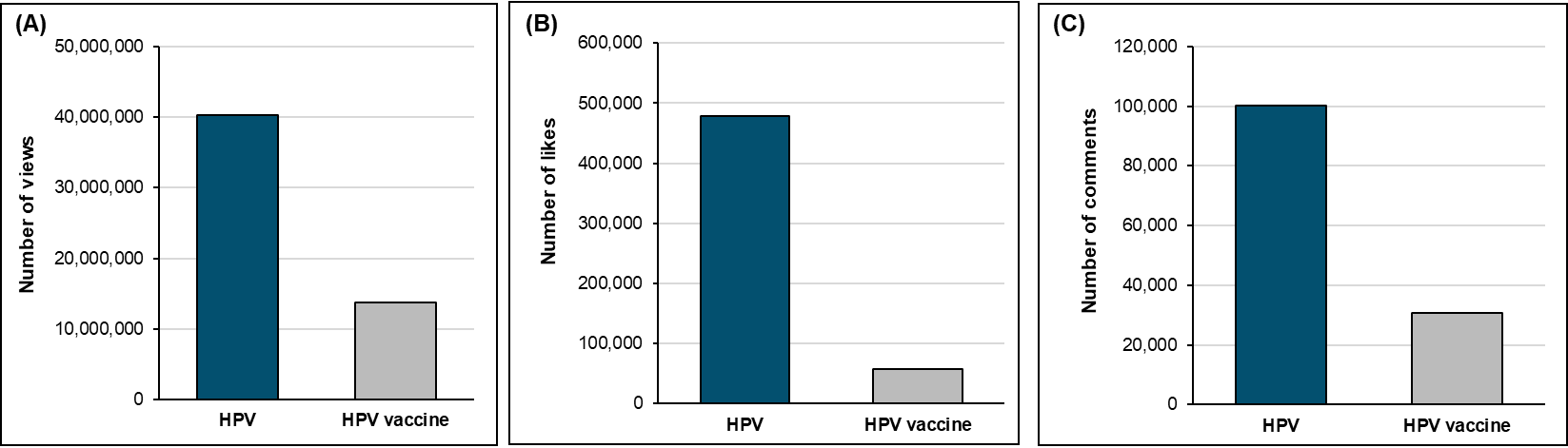
**

Figure illustrates data on public’s interest in HPV- and HPV vaccine-related videos on YouTube. **PANEL A** shows lifetime views on HPV and HPV vaccine-related videos on YouTube. **PANEL B** illustrates the total number of likes on HPV and HPV vaccine-related content on YouTube. **PANEL C** includes the total number of comments on HPV and HPV vaccine-related videos.

**Abbreviations:** HPV, Human papillomavirus

*YouTube API data was abstracted to determine audience engagement including the total number of views, likes, and comments on the top 500 YouTube videos with HPV and HPV vaccine content.
