# Supplementary figures and images for: Consumption of Health-Related Videos and Human Papillomavirus Awareness: Cross-Sectional Analyses of a US National Survey and YouTube From the Urban-Rural Context
Source: J Med Internet Res. 2024 Jan 15;26:e49749. doi: 10.2196/49749 (PMC10825763; doi:10.2196/49749)

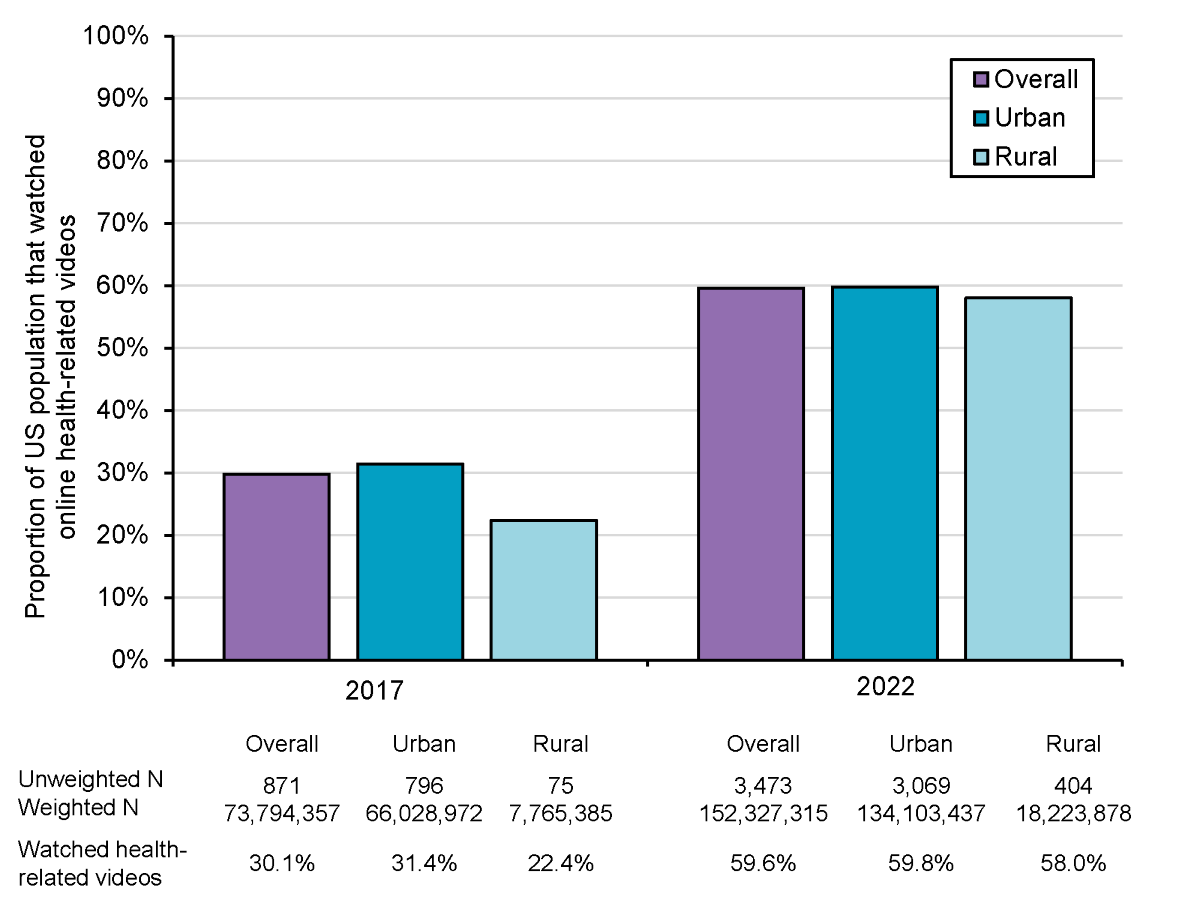

Supplement: Multimedia Appendix 2 [file jmir_v26i1e49749_app2.png]

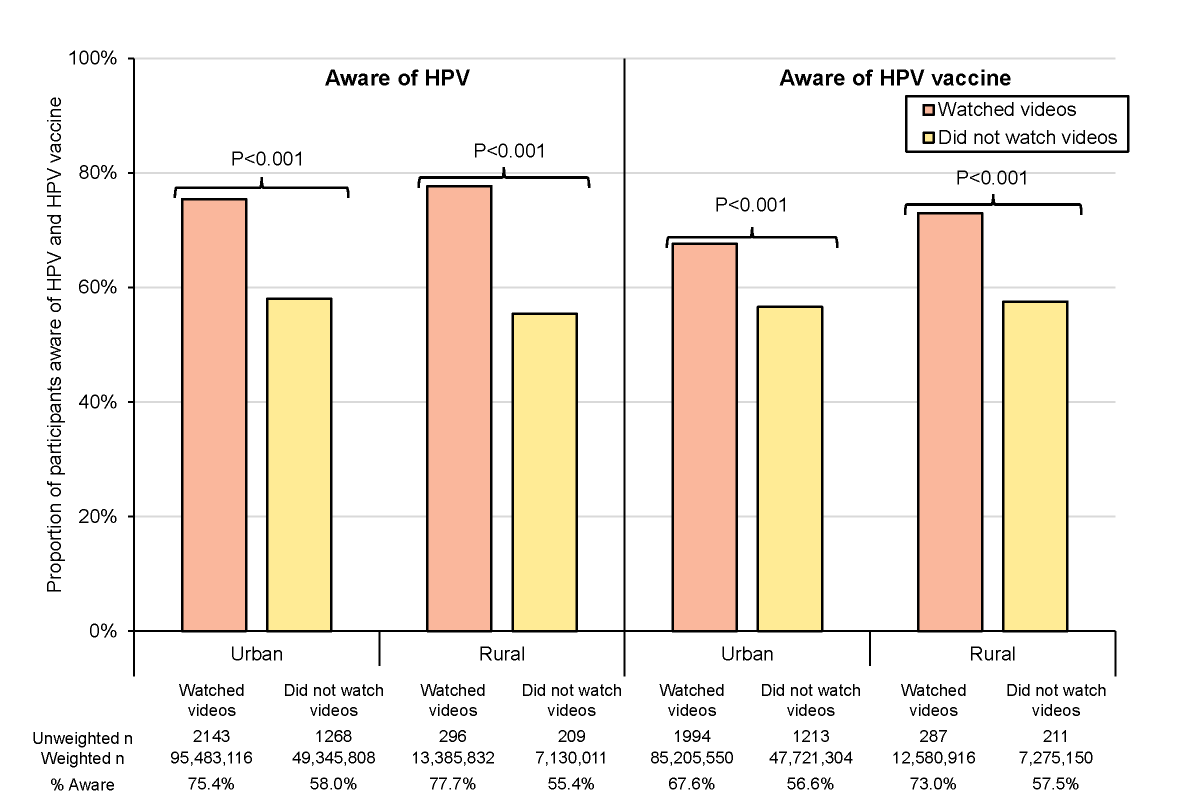

Supplement: Multimedia Appendix 3 [file jmir_v26i1e49749_app3.png]
